# Circulating small extracellular vesicles in chronic kidney disease and vascular calcification: “Tiny packages with big biological mission”

**DOI:** 10.1007/s10157-025-02793-7

**Published:** 2025-12-16

**Authors:** Shintaro Mandai

**Affiliations:** Department of Nephrology, Graduate School of Medical and Dental Sciences, Institute of Science Tokyo, Tokyo, Japan

**Keywords:** Small extracellular vesicles, Chronic kidney disease, Vascular calcification, Inter-organ communication, Renal senescence

## Abstract

Chronic kidney disease (CKD), a condition affecting over 850 million people worldwide, is a major global health issue. CKD leads to multimorbidities and disease complexity, expediting the aging process and increasing mortality rates—a phenomenon sometimes referred to as “renal senescence.” The leading cause of death in patients with CKD is cardiovascular disease (CVD), accounting for one-third to one-half of all deaths, in stark contrast to cancer, which is the primary cause of death in the general population. While previous studies on kidney disease have focused extensively on urinary extracellular vesicles due to their potential as non-invasive diagnostic tools and their origin from kidney cells, our research highlighs the significance of circulating small extracellular vesicles (cEVs). We demonstrated that cEVs act as key mediators in the pathological intercellular and inter-organ communication between the kidneys and vascular smooth muscle cells (VSMCs). However, the biogenesis, cargo, and biological functions of cEVs remain incompletely understood under physiological and pathological conditions, including CKD. We identified microRNA (miRNA) transcriptomic signatures encapsulated in cEVs from CKD animal models, which were validated in human CKD samples. Notably, the depletion of specific miRNAs in CKD-derived cEVs promoted osteogenic differentiation of VSMCs and the deposition of calcium-phosphate crystals in vessels. In contrast, miRNAs enriched in cEVs from healthy individuals suppress these pathological processes, acting as a safeguard. These findings and future research could pave the way for the development of diagnostic and therapeutic platforms leveraging cEVs in nephrology.

**Graphical Abstract:**

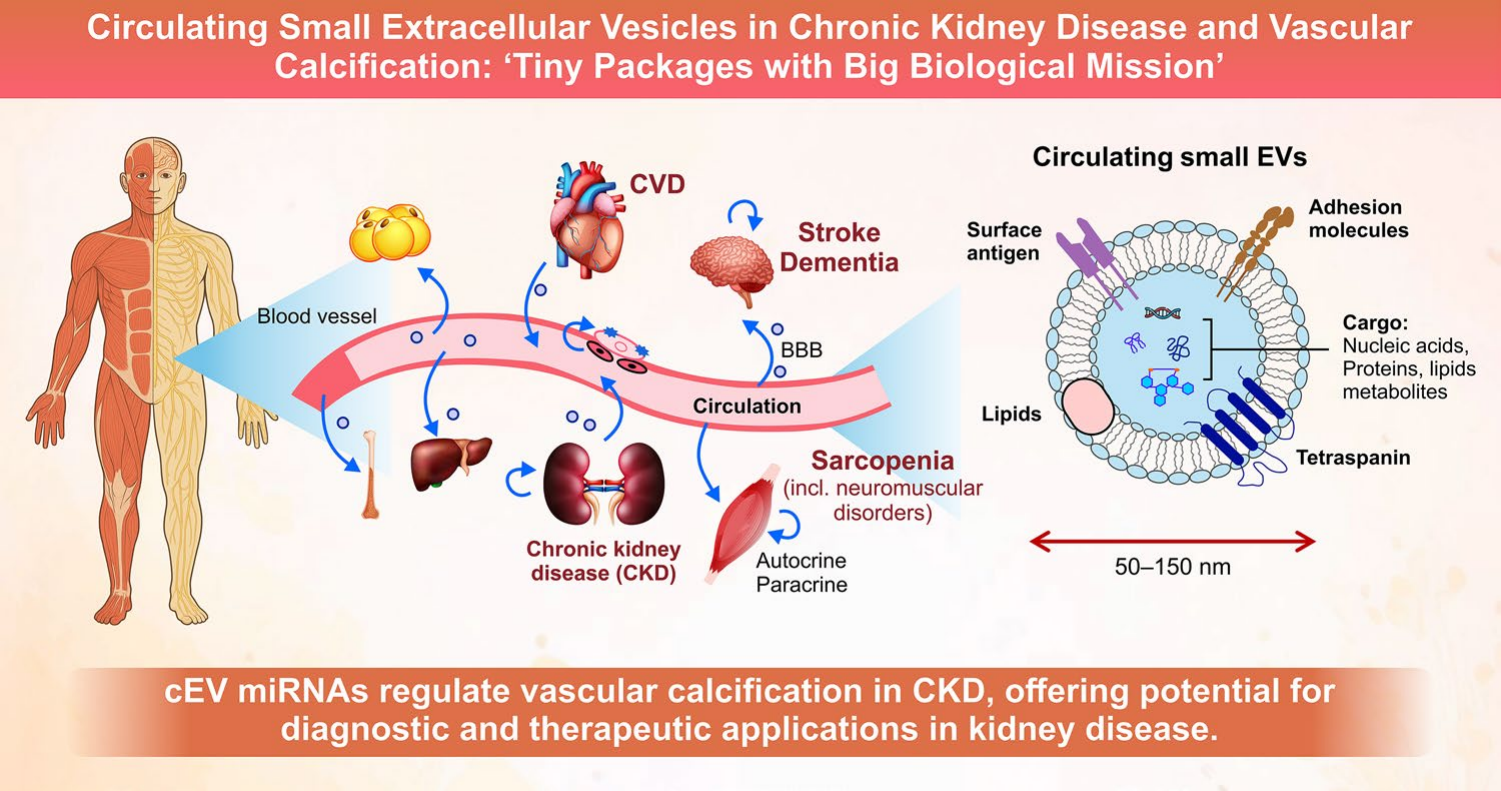

## Introduction

Chronic kidney disease (CKD) is characterized by decreased glomerular filtration rate (GFR) and abnormalities in urinalysis findings or kidney structure that persist for at least 3 months [1]. The majority of CKD patients experience a progressive and irreversible decline in kidney function. CKD etiology and pathogenesis are complex, and there are currently no treatment strategies that effectively target the underlying pathogenesis to halt or reverse kidney function loss. Diabetes mellitus and hypertension, the primary causes of dialysis-dependent CKD, have elevated the latter to a significant global health issue, affecting over 850 million people worldwide [2]. If the increasing trends of CKD prevalence and population aging observed in 1990–2016 continue, the proportion of individuals with CKD categories G3–G5 is projected to surpass 10% in many parts of the world by 2050 [3].

Kidneys regulate body fluid volume and osmolarity, eliminate waste products, and function as endocrine organs to maintain the homeostasis of multiple systems (e.g., bone mineralization, hematopoiesis, and blood pressure regulation). Thus, kidney function decline alters the composition of biofluids, particularly serum and plasma. The alteration manifests clinically as electrolyte and acid–base disorders, chronic kidney disease—mineral and bone disorder (CKD-MBD), and uremia. Therefore, CKD causes various complications from its early stages in remote organ systems.

**Fig. 1:**
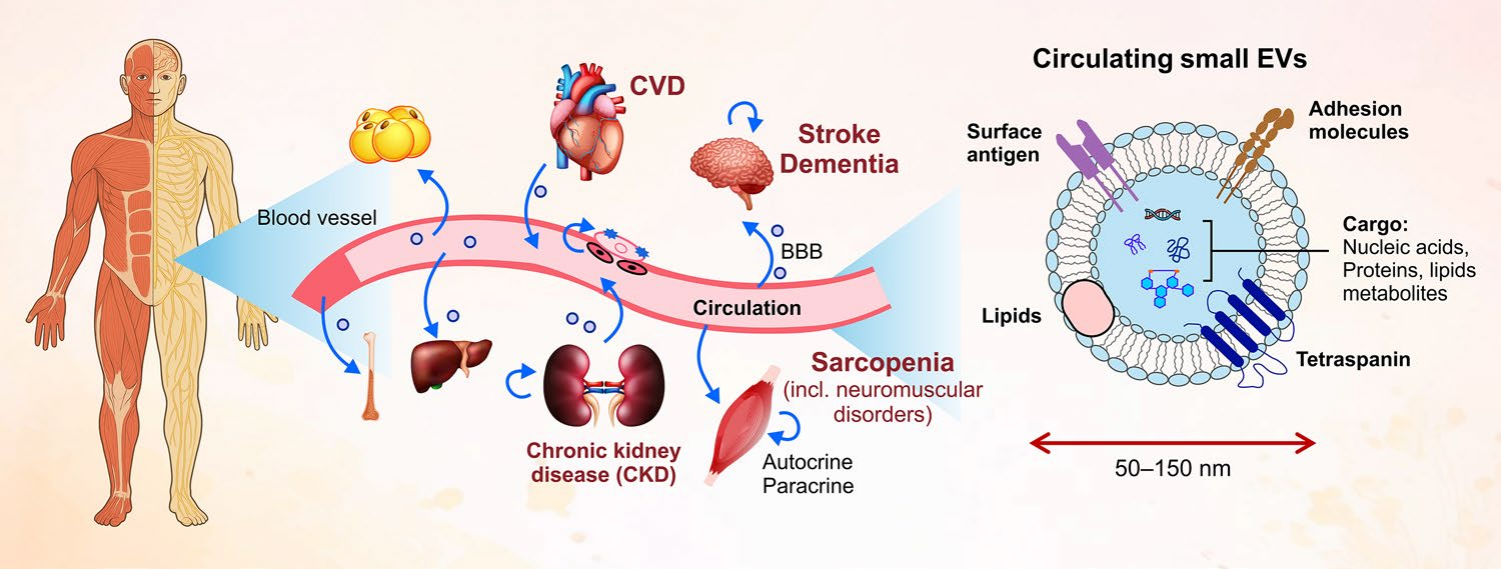
Schematic representation of circulating extracellular vesicles (cEVs) and their potential roles in CKD-related multi-morbidities and premature aging The left panel illustrates a human body with a focus on the circulatory system, highlighting the kidney affected by CKD and other organs, such as the heart, blood vessels, vascular endothelial cells (VSMCs), skeletal muscle, brain, and bone. The right panel provides a schematic representation of cEVs, which are nanosized particles (50–150 nm) enclosed by a lipid bilayer membrane. cEVs contain various general molecules, including proteins, lipids, and nucleic acids, and are characterized by the presence of tetraspanins on their surface. cEVs are depicted as potential mediators of autocrine and paracrine communication, forming an intricate network of intercellular and inter-organ interactions. These interactions may contribute to CKD-associated multi-morbidities and, ultimately, premature aging, a phenomenon known as “renal senescence.” *CKD* Chronic kidney disease, *CVD* cardiovascular disease

Cardiovascular disease (CVD) is the major complication of CKD and the leading cause of CKD-related death. One-third to one-half of patients with advanced CKD die from cardiovascular causes. CKD contributed to the estimated 17.9 million deaths from CVD in 2019, accounting for 32% of global mortality [4]. Patients with CKD are also susceptible to infectious diseases [5], cognitive impairment [6, 7], frailty and sarcopenia [8], and fractures [9]. Tonelli M et al. [10] previously reported that patients seen by nephrologists are the most complex ones, based on the nine-disease complexity scoring, which includes comorbidities, polypharmacy, and mental disorders. These systemic problems exacerbate kidney dysfunction [11–13], creating a vicious cycle that positions the kidney as a central hub organ in the body (Fig. 1). Tian YE et al. demonstrated that CKD was the chronic disease that most commonly yields the negative gap between an organ’s biological and chronological ages [14]. However, the pathogenesis of multimorbidity and premature aging [15], sometimes referred to as “renal senescence,” which may represent pathological kidney aging, cannot be fully explained by conventional circulating substances associated with CKD-MBD or uremic toxins. This gap suggests the involvement of unconventional mediators.

This review focuses on the emerging role of extracellular vesicles (EVs), particularly circulating small extracellular vesicles (cEVs), as mediators of inter-organ communication between the kidney and other body organs (Fig. 1). This review provides an overview of the biogenesis and potential roles of cEVs as disease biomarkers and therapeutic targets in kidney disease and vascular calcification (VC).

### Current definition and understanding of EVs

Almost all prokaryotic and eukaryotic cells produce and release EVs into the extracellular environment. EVs are biological nanoparticles composed of a lipid bilayer membrane that encapsulates molecules such as proteins, lipids, nucleic acids, and metabolites [16] (Fig. 1), which are shielded from degradation and can be delivered to recipient cells. EVs mediate paracrine and endocrine signaling by transferring their cargo to recipient cells, thereby modulating gene expression and molecular pathways in the recipient cells.

**Fig. 2:**
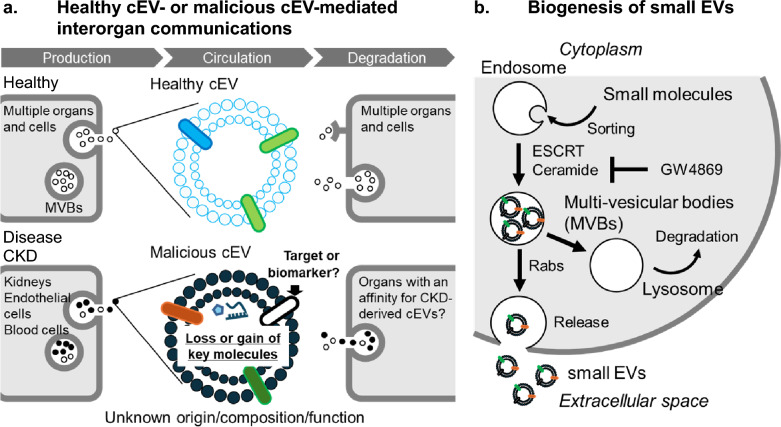
Biogenesis of small extracellular vesicles (small EVs) This is a schematic illustration of the process of small EV biogenesis, from the cytoplasm to the extracellular space. Small EVs originate from the inward budding of the endosomal membrane, forming multi-vesicular bodies (MVBs) that contain intraluminal vesicles (ILVs). MVBs can either fuse with lysosomes for degradation or with the plasma membrane to release ILVs as small EVs into the extracellular space. The first step of this process involves ceramide synthesis, which is critical in ILV formation. The inhibition of ceramide synthesis is a potential strategy to block small EV biogenesis

EVs were first reported in 1967 by Wolf P, who described them as “platelet dust” [17]. These vesicles, which could be separated by ultracentrifugation from fresh plasma devoid of intact platelets, were rich in phospholipids and exhibited coagulant properties similar to those of Platelet Factor 3. In 1981, Trams EG et al. [18] referred to EVs as “microvesicles,” which were identified in culture media of normal and neoplastic cell lines. Using electron microscopy, they identified two populations of vesicles: one with an average diameter of 500–1,000 nm and another measuring approximately 40 nm in diameter. Shortly thereafter, Harding C et al. [19] and Pan BT et al. [20] reported the secretion of small vesicles (~ 50 nm in diameter) of endocytic origin from cultured reticulocytes. They observed that these small vesicles were found inside large multi-vesicular endosomes, which fused with the plasma membrane to release the internal vesicles into the extracellular space (Fig. 2). In 1987, Johnstone RM et al. [21] named these reticulocyte-derived vesicles “exosomes,” which were purified by centrifugation at 100,000 g for 90 min.

These historical findings led to the classification of EVs into biogenesis-based subtypes, including plasma membrane-derived “microvesicles” or “ectosomes” (typically 100–1,000 nm) and endosome-origin “exosomes” (50–150 nm) [22]. Other EV types include “exomeres” (< 50 nm), “apoptotic bodies” (1000–5,000 nm), “migrasomes” (500–3,000 nm), and “oncosomes” (1000–10,000 nm). However, to the best of our knowledge, no universal markers exist to classify these subtypes [22]. For example, among the tetraspanins CD63, CD9, and CD81, which are considered putative “exosome” markers, CD9 and CD81 are often enriched on ectosomes [22]. In addition, ectosome size overlaps with exosome size, making this parameter alone insufficient for subgroup classification. Consequently, inconsistent definitions, purification methods, and characterization techniques have made it challenging to accurately interpret EVs’ proper fractions and functions in many previously published studies. The MISEV guidelines recommend using the generic term “extracellular vesicles” instead of subtype-specific terms to address this issue.

### Biogenesis of small EVs and potential transfer to recipient cells

Most small EVs originate from late endosomes through the inward budding of the endosomal membrane, which forms intraluminal vesicles (ILVs) within multi-vesicular bodies (MVBs) [23, 24] (Fig. 2). In this first step, the endosomal sorting complex required for transport (ESCRT)-dependent and ESCRT-independent machinery regulates ILV formation and release [25]. The ESCRT system includes four complexes (ESCRT-0, -I, -II, and -III) that recognize and sort ubiquitinated proteins into ILVs. ESCRT-independent pathways for the biogenesis of small EVs include a neutral sphingomyelinase 2 (nSMase2)-ceramide-dependent pathway [26]. The enzyme nSMase2 is essential for producing ceramide, which induces membrane curvature and promotes ILV formation. Several other proteins, such as components of lipid rafts, also contribute to ESCRT-independent ILV formation. For example, the syndecan-syntenin-ALIX complex can bypass the early ESCRT machinery and directly influence small EV formation. At the same time, CD63 can promote ILV formation through both ESCRT- and ceramide-independent mechanisms. However, only blocking the ceramide-dependent pathway with GW4869 [27] (Fig. 2), a noncompetitive neutral sphingomyelinase inhibitor, can prevent the biogenesis of small EVs in vitro [28] and in vivo [29].

After MVB formation, some MVBs fuse with lysosomes and are degraded before fusing with the cytoplasmic membrane (Fig. 2). Other MVBs fuse with the plasma membrane, releasing ILVs into the extracellular space, thereby completing the biogenesis of small EVs. Several mechanisms also control this second step in small EV biogenesis. Ostrowski M et al*.* [30] demonstrated that Rab2b, Rab9a, Rab5a, Rab27a, and Rab27b—but not Rab7, Rab11a, or other Rabs—are involved in small EV secretion from HeLa cells, using RNA interference. In addition to Rabs, lysosome-associated protein transmembrane 4B (LAPTM4B) and Hsp90 promote MVB transport toward the plasma membrane and small EV secretion [31, 32]. Depending on the cell type, multiple mechanisms might act in parallel and be responsible for the secretion of different cargos or subpopulations of MVBs.

The packaging of cargo into small EVs is selective and tightly regulated, involving mechanisms that are not yet fully elucidated. The nucleic acids encapsulated in EVs range from complete messenger RNA (mRNA), fragments of mRNA, long noncoding RNA (lncRNA), microRNA (miRNA), ribosomal RNA (rRNA), single-stranded DNA (ssDNA), and mitochondrial DNA (mtDNA), to double-stranded DNA (dsDNA). In 2007, Valadi et al. [33] provided the first known evidence of the presence of miRNAs within small EVs and the intercellular transfer of mRNAs and miRNAs. They demonstrated that RNAs from the MC/9 human mast cell line, labeled with 3H-uracil, were transferred to CD4, MC/9, and HMC-1 cells, modulating recipient cell protein production and gene expression [33]. miRNAs are short (18–25 nucleotides) noncoding RNAs that suppress protein production by either blocking translation or inducing deadenylation, leading to target mRNA degradation [34]. Regarding evidence of in vitro intercellular miRNA transfer, Zomer A et al. [35] used the Cre-LoxP system and intravital imaging in living mice to show local and systemic EV-mediated communication between tumor cells. Another study established a CRISPR-Cas9-based reporter system known as CROSS-FIRE, enabling single-cell resolution detection of small EV-mediated functional RNA transfer [36].

Despite many advances in understanding small EV biogenesis, several challenges persist in studying cEVs, particularly in vivo. Accurate tracking of cEVs in living organisms is limited by their nanoscale size, rapid clearance, and the lack of standardized, high-specificity labeling techniques. Common fluorescent dyes often produce nonspecific signals or aggregate, while optical imaging faces limited tissue penetration and high background interference. In addition, the absence of universally accepted EV isolation methods, together with variability in yield, purity, and reproducibility, complicates downstream analyses. The heterogeneity of EV populations and variability in cargo content further hinder biomarker discovery and functional interpretation. Overcoming these challenges will require the development of robust imaging platforms, standardized protocols, and integrative analytical approaches.

### Roles of urinary EVs in kidney disease

Urine analysis is a non-invasive and more accessible modality than the analysis of other body fluids. Numerous studies have analyzed urinary EVs (uEVs) and their contents and their potential roles as disease diagnosis or severity biomarkers. In contrast to the diverse origins of cEVs, uEVs are predominantly produced by cells in the urinary tract [37]. uEVs express typical apical membrane proteins, including channels and transporters; however, they do not express basolateral proteins. The intact glomerular filtration barrier allows the passage of structures measuring ≤ 4 nm. Thus, most cEVs cannot bypass filtration via healthy glomeruli and do not leak into the urinary tract. According to previous research, 5,090 out of 5,113 identified uEV proteins (99.96%) expressed RNA within the epithelium of the urinary tract—including the kidney, urinary bladder, and, in males, the testis, prostate, epididymis, and seminal vesicle [38].

Since the discovery of small EV-mediated cargo transfer between cells [33], Street JM et al. reported the potential for in vitro intratubular communication via small EVs purified by ultracentrifugation, using murine cortical collecting duct (mCCDc11) cell lines [39]. Aquaporin-2 (AQP2) was detected in small EVs that were stimulated with desmopressin for 48 h, but not in the ones that were released by unstimulated cells. Subsequently, this group found that cells exposed to small EVs derived from desmopressin-stimulated cells expressed AQP2 after 48 h of co-culture, and the induced AQP2 expression was functional. Gildea JJ et al. [40] demonstrated that distal tubule and collecting duct cell lines could uptake fluorescently labeled small EVs released from CD9-RFP and CD63-EGFP human renal proximal tubule cell lines in vitro. Hunter RW et al. [41] further reported the potential for RNA transfer from podocytes to renal tubular epithelial cells in mice under physiological conditions, using a method to track extracellular podocyte RNA with SLAMseq (SH-linked alkylation for the metabolic sequencing of RNA in tissue). To summarize and contrast the key features of these two EV populations, Table 1 presents a comparative overview of uEVs and cEVs, including their cellular origins, molecular contents, and clinical relevance in kidney disease.

**Table 1:** Comparative summary of urinary and circulating extracellular vesicles (uEVs and cEVs): origins, molecular contents, and clinical relevance in kidney disease

Characteristic	Urinary extracellular vesicles	Circulating extracellular vesicles
Origin	Predominantly from kidney and urinary tract cells (e.g., podocytes and tubular epithelial cells)	Broad range of tissues and organs (e.g., the kidney, endothelial cells, and immune cells)
Biological fluid	Urine	Blood (plasma/serum)
Cargo	Kidney-specific proteins (e.g., aquaporins, and transporters), miRNAs, and lncRNAs	Systemic proteins, miRNAs, lncRNAs, and metabolites
Biological role	Local intercellular communication within the urinary tract	Mediators of systemic inter-organ communication, including kidney-to-vascular signaling
Clinical implications	Noninvasive biomarkers for kidney-specific diseases (e.g., diabetic nephropathy and IgA nephropathy)	Potential biomarkers and therapeutic targets for systemic complications of kidney disease, such as vascular calcification
Advantages	Easily accessible and non-invasive for sampling	Reflects systemic pathophysiological changes
Challenges	Normalization issues due to variations in urine output and urine concentration	Heterogeneity in origin and cargo composition
Potential applications	Diagnostic biomarkers for kidney diseases	Diagnostic biomarkers and therapeutic targets for systemic complications (e.g., vascular calcification)

Numerous studies have attempted to apply miRNAs within uEVs as biomarkers of kidney diseases. A study of 32 patients with CKD showed that miR-29c expression was decreased, and miR-200b was increased in uEVs compared to healthy individuals [42] (Table 2). Another research group reported that miR-29c expression correlated with eGFR and tubulointerstitial fibrosis among 25 uEV samples, including 15 patients with CKD [43]. This miRNA was also reduced in uEVs from 18 patients with IgA nephropathy [44]. A previous study analyzed uEVs from 12 patients with type 1 diabetes mellitus (DM1) and persistent microalbuminuria, and 12 normoalbuminuric DM1 patients [45]. uEVs from microalbuminuric patients showed increased miR-130a and miR-145, but decreased miR-155 and miR-424. Consistently, miR-145 levels were elevated in the uEVs and glomeruli of mice with a diabetic kidney disease model.

**Table 2:** Summary of kidney disease-related miRNAs in extracellular vesicles and tissue-specific roles of miR-16-5p and miR-17 ~ 92 cluster

Kidney disease subtype	EV subtype	Origin of EVs or miRNAs	miRNAs	Function and role	Purification method	Reference
miRNAs within urinary EVs						
Chronic kidney disease	Small EVs	Urine	miR-29a, miR-29c, miR-200 family	Diagnostic role for tubulointerstitial fibrosis (all downregulated)	Ultracentrifugation	Lv et al. [42]. Am J Physiol Renal Physiol 2013;305(8):F1220-7
Chronic kidney disease	Small EVs	Urine	miR-29c/miR-21	Diagnostic role for fibrosis (down/up). miR-29c may mitigate fibrosis	Polyethylene Glycol (PEG) Precipitation	Lv et al. [63]. Exp Mol Pathol 2018;105(2):223–228, Lv et al. [64]. Int Urol Nephrol 2018;50(5):973–982
Diabetic kidney disease	Small EVs	Urine	miR-192	Diagnostic role for microalbuminuria (up)	Ultracentrifugation	Jia et al. [65]. J Diabetes Res. 2016;2016:7,932,765
Chronic kidney disease	Small EVs	Urine	miR-181a	Diagnostic role for CKD (down). miR-181a may mitigate glomerulosclerosis, tubular epithelial injury, and inflammation	Ultracentrifugation	Khurana et al. [43]. RNA 2017;23(2):142–152, Liu et al. [66]. Nephron 2022;146(6):637–646. Liu et al. [67]. Mol Med. 2018;24:49
Diabetic kidney disease	Small EVs	Urine	miR-15b/miR-34a/miR-636	Diagnostic role for diabetic kidney disease (all upregulated)	Spin column-based exosome RNA isolation (Norgen Biotek kit)	Eissa et al. [68]. J Diabetes Complications 2016;30(8):1585–1592
Lupus nephritis	Small EVs	Urine	miR-21/miR-150/miR-29c	Early detection of renal fibrosis and end-stage kidney disease risk prediction (up/down/down)	Polymer-based precipitation using miRCURY™ Exosome Isolation Kit (Exiqon)	Solé et al. [69]. Cells 2019;8(8):773
IgA nephropathy	Small EVs	Urine	miR-215-5p/miR-378i/ miR-29c/miR-205-5p	Non-invasive biomarkers for IgA nephropathy diagnosis (up/up/down/down)	Ultracentrifugation	Min et al. [44]. J Clin Lab Anal 2018;32(2):e22226
Diabetic kidney disease (type 1 diabetes)	Small EVs	Urine	miR-130a/miR-145/ miR-155/miR-424	Early biomarker candidates for incipient diabetic nephropathy (up/up/down/down)	Ultracentrifugation	Barutta et al. [45]. PLoS One 2013;8(11):e73798
miRNAs within circulating EVs						
Experimental kidney injury in Sprague–Dawley rats	Small EVs	Plasma	let-7c-5p, miR-532-3p, miR-429, miR-143-3p, miR-770-5p, miR-224-5p, miR-29b-3p, let-7a-5p	Biomarker candidates for fibrosis and kidney injury pathways. Mostly harmful (fibrosis, ECM modulation)	Polymer-based precipitation using Total Exosome Isolation Kit (Invitrogen)	Xie et al. [70]. Gene 2017;627:1–8
Diabetic kidney disease	Small EVs	Plasma and Urine	miR-155-5p/miR-34a-5p/ miR-30a-5p/let-7b-5p	Regulation of fibrosis-related pathways; metabolic network analysis for diabetic kidney disease (down/down/down/up)	Ultracentrifugation for urine and ExoQuick™ precipitation for plasma (System Biosciences)	Park et al. [71]. Genomics, 2022;114(4):110407
Diabetic kidney disease	Small EVs	Serum	miR-1246, miR-642a-3p, let-7c-5p, miR-1255b-5p, let-7i-3p, miR-5010-5p, miR-150-3p, miR-4449	Associated with albuminuria; MAPK and integrin signaling; diagnostic potential (all upregulated)	ExoQuick™ precipitation (System Biosciences)	Kim et al. [52]. J Transl Med, 2019;17(1):236
FSGS and MCD	Small EVs	Plasma and Urine	Plasma miR-30b/c, miR-34b/c, miR-342; urinary miR-1225-5p, miR-1915, miR-663, miR-155	Differential diagnosis between FSGS and MCD; biomarker potential (up/up/up/up/down/down/up)	Exosome RNA Isolation Kit (Norgen Biotek)	Ramezani et al. [53]. Eur J Clin Invest 2015;45(4):394–404
Chronic kidney disease	Small EVs	Serum	miR-16-5p, miR-17-5p, miR-20a-5p, miR-106b-5p	Vascular calcification regulation via VEGFA signaling; biomarker and therapeutic target (all downregulated)	Polymer-based precipitation using Total Exosome Isolation Kit (Invitrogen)	Koide et al. [57]. Circ Res 2023;132(4):415–431
Kidney tissue miRNAs						
Sepsis-associated AKI in C57BL/6 mice	NA	Renal tubular epithelial cells	miR-16-5p	Harmful: promotes apoptosis, inflammation, and renal injury; aggravates AKI	NA	Li et al. [72]. Ren Fail. 2024;46(1):2322688
Ischemia/reperfusion AKI in C57BL/6 mice	NA	Renal tubular epithelial cells	miR-16-5p	Harmful: overexpression increases kidney apoptosis, worsens function, targets BCL2	NA	Chen et al. [73]. Sci Rep 2016:6:27945
Diabetic kidney disease model in Sprague–Dawley rats	Small EVs	Human urine-derived stem cells (recipient cells: podocytes	miR-16-5p	Protective: overexpression protects podocytes, suppresses VEGFA	ExoQuick™ precipitation (System Biosciences)	Duan et al. [74]. J Cell Mol Med 2021;25(23):10798–10813
Ischemia/reperfusion AKI in C57BL/6 mice	NA	Renal tubular epithelial cells (mouse model)	miRNA-17-5p	Protective: targets Death Receptor 6 (DR6), suppresses apoptosis; induced by p53	NA	Hao et al. [75]. Kidney Int 2017;91(1):106–118
Ischemia–reperfusion injury AKI, sex differences in C57BL/6 mice	NA	Renal tubular epithelial cells (mouse and human)	miR-17 ~ 92 cluster	Protective (female-biased): suppresses JAK/STAT3 signaling, reduces inflammation and EMT	NA	Suda et al. [76]. Physiology 2024;39(S1):1516

*down* downregulated, *up* upregulated, *AKI* acute kidney injury, *CKD* chronic kidney disease, *ECM* extracellular matrix, *FSGS* focal segmental glomerulosclerosis, *MCD* minimal change disease; *miRNA* microRNA, *NA* not applicable, *VEGFA* Vascular endothelial growth factor A

The major pitfall in analyzing uEVs is that normalization is challenging when comparing uEV biomarkers between individuals. Kidney function and volume are associated with renal uEV excretion [46]. When uEV excretion was measured before and after human kidney donor nephrectomy and rat nephrectomy, nephrectomy reduced uEV excretion less than expected, given the compensatory hypertrophy of the remaining kidney. In addition, due to the heterogeneity in uEV analyses, despite various promising findings, readiness for clinical translation awaits the reproduction of these findings in independent large cohorts.

### Known and emerging roles of circulating EVs in kidney disease

As mentioned in the previous section, the spaces where cEVs circulate and where uEVs exist in the urinary tract are almost entirely isolated. The origin of cells and their heterogeneity differ between cEVs and uEVs. cEVs originate from a broader range of tissues than uEVs. Thus, elucidating the origin, contents, recipient cells, and functions of cEVs after reception remains challenging.

As far as we can tell, there is limited evidence on the biological signal transduction of cEV contents to the kidneys. However, previous research has demonstrated that the heart receives cEVs from various other organs, including the kidneys [47]. To obtain perfusates containing soluble secreted factors and small EVs present within the intercellular space of the murine heart, this group used collagenase II to hydrolyze the extracellular matrix and loosen cardiac tissue structure. They freshly isolated intact mouse hearts, perfused them with a perfusion medium, and finally separated small EVs from soluble secreted factors after the differential centrifugation of these perfusates. They performed a proteomic analysis of heart-small EVs and identified proteins, such as kielin/chordin-like protein, presumably originating from the kidneys. Another study found that human placenta-derived small EVs purified with ultracentrifugation carry anti-angiogenic factors that might cause proteinuria via glomerular endothelial dysfunction [48].

Several studies have demonstrated the possible involvement of EVs in the pathogenesis of autoimmune disease-associated kidney dysfunction. In these studies, the majority of analyzed microparticles were separated from serum or plasma using flow cytometry, which seems to be classified under the “microvesicles.” A previous study showed that the plasma concentration of EVs expressing myeloperoxidase (MPO) is increased in patients with ANCA-associated vasculitis (AAV) [49]. MPO-positive EVs expressing inflammatory biomarkers such as PTX3 and HMGB1 were associated with disease activity in AAV. Patients with AAV also displayed more platelet-derived EVs, expressing higher levels of chemokines, adhesion molecules, growth factors, and apoptotic factors than healthy individuals [50]. Plasma EV levels were correlated with AAV activity, elevated serum creatinine, and higher glomerular histologic damage, including interstitial infiltration, interstitial fibrosis, and tubular atrophy.

To our knowledge, scientific evidence on the contents and biological functions of cEVs is minimal, both in CKD animal models and in patients. To provide a structured overview of current findings, Table 2 summarizes published studies reporting kidney disease-related microRNAs identified in extracellular vesicles derived from plasma, serum, or urine. The table includes information on EV subtypes, cellular origins, purification methods, and the functional roles of specific miRNAs, with a particular focus on miR-16-5p and the miR-17 ~ 92 cluster, whose roles will be elaborated in subsequent sections of this review. A previous study selectively analyzed the expression of miR-21, miR-142-3p, and miR-221 in renal histology with 34 patients with Banff classification-based high and low fibrosis scores in post-kidney transplantation [51]. The expression of miR-21 in plasma small EVs, precipitated with 24% polyethylene glycol and showing a mean diameter of 131 nm, was increased among patients with higher grade interstitial fibrosis and tubular atrophy. In another study, RNA sequencing of miRNAs was performed in small EVs isolated using a polymer-based precipitation method [52]. This study identified eight miRNAs uniquely upregulated in patients with diabetic kidney disease compared to healthy volunteers and diabetic patients without nephropathy. Among these, miR-4449 was consistently upregulated in patients with diabetic kidney disease and correlated with the degree of albuminuria, suggesting its potential as a biomarker for DN. In another study, a miRNA array-based screen of plasma small EVs from patients with primary focal segmental glomerulosclerosis (FSGS, *n* = 16), minimal change disease (MCD, *n* = 5), and healthy controls was performed [53]. This study found that miR-30b, miR-30c, miR-34b, miR-34c, and miR-342 were upregulated in patients with MCD compared to those with FSGS and healthy controls.

Previous studies have demonstrated that VSMC-derived small EVs play a dual role in the regulation of VC and coagulation [54]. Prothrombin, a vitamin K-dependent protein, inhibits small EV-mediated calcification via Gla domain interactions with phosphatidylserine and contributes to thrombogenesis. Thus, warfarin treatment may accelerate VC by impairing the carboxylation of these coagulation proteins. However, the pathogenic link between small EVs and VC in the context of CKD remains unclear.

### Mechanism of circulating small EV-driven vascular calcification in CKD

**Fig. 3:**
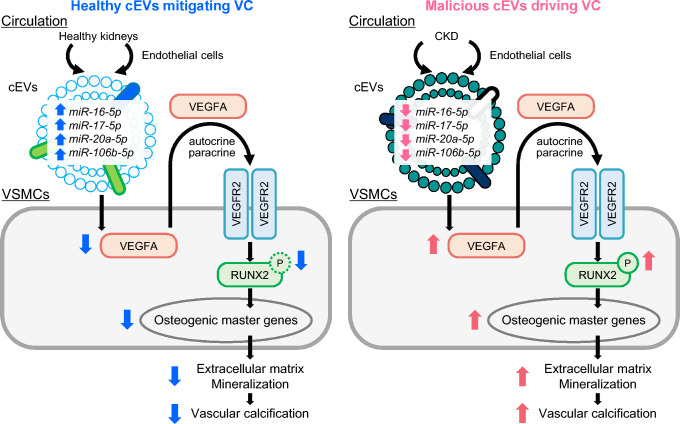
Inter-organ communication between CKD-affected kidneys and vascular smooth muscle cells (VSMCs) in vascular calcification Adapted and modified from the graphical abstract of Ref. [57], this schematic representation illustrates the role of CKD-derived circulating small extracellular vesicles (cEVs) in the promotion of vascular calcification (VC). CKD-derived cEVs exhibit a pathogenic property by promoting calcium deposition in VSMCs due to alterations in their miRNA profiles. Specifically, CKD-derived cEVs are depleted of protective miRNAs, including miR-16-5p, miR-17-5p, miR-20a-5p, and miR-106b-5p, which are typically enriched in healthy cEVs. These miRNAs cooperatively target VEGFA (vascular endothelial growth factor A), suppressing its mRNA and protein expression in VSMCs. The loss of this suppression in CKD-derived cEVs leads to VEGFA-VEGFR2 signaling activation, which facilitates osteogenic marker gene transcription, phenotypic switching of VSMCs, and vascular calcification. This figure highlights the protective role of miRNAs in healthy cEVs against VC (left) and the pathological suppression of this safeguard mechanism in CKD (right) Abbreviations: CKD, chronic kidney disease; miRNA, microRNA

VC is a fundamental issue underlying CVD pathogenesis in CKD. VC is characterized by calcium phosphate crystal deposition in blood vessels [55]. VSMCs constitute the cellular elements of the medial layer of blood vessels. The process of osteogenic differentiation from contractile VSMCs into non-contractile VSMCs, referred to as the phenotypic switching of VSMCs, leads to extracellular matrix deposition, matrix mineralization, and ectopic calcification [56]. However, this process’s pathogenesis and key signaling molecules are not fully understood. Our recent research has focused on inter-organ communication between the kidneys and remote VSMCs [57] (Fig. 3).

Our research group first revealed that serum from CKD model rats, established with 0.75% adenine for 4 weeks, exhibited properties that drive the phenotypic switching of VSMCs and calcification [57]. In the A10 clonal embryonic rat aortic smooth muscle cell line exposed to a calcifying medium containing 4.0 mM inorganic phosphate (Pi), rat CKD serum at 5% concentration in the medium increased calcified deposits and calcium concentrations, as shown by Alizarin red staining. The transcription of osteogenic marker genes, including *OSX* (osterix), *OCN* (osteocalcin), *OPN* (osteopontin), and *MGP* (matrix gla protein), was upregulated in cells treated with high-Pi and CKD serum. According to a previous report, serum from patients on chronic hemodialysis increased calcium deposition in VSMCs compared to that from dialysis-independent patients [58]. However, the mechanisms and key components in CKD serum responsible for this effect were unidentified.

We found that a fraction of small EVs within the serum, but not small EV-depleted serum, was responsible for the increased calcified deposits, calcium levels, and transcription of osteogenic gene markers. Polymer-based purification was primarily used in these experiments to achieve > 90% cEV recovery, enabling subsequent functional assays of cEVs and cEV-depleted serum and translation to human diagnostic workups [59, 60]. We observed similar size distributions between control rat small EVs and CKD small EVs. Cells were treated with 5% serum or 50 μg of cEVs (in protein content), which was equivalent to the amount extracted from 5% serum using polymer-based purification. Small EVs from CKD rats, which were purified via differential ultracentrifugation, facilitated VC dose-dependently in a high-Pi milieu.

These findings, derived from in vitro assays performed at physiological serum EV concentrations, demonstrated that cEVs propagate unconventional signaling molecules between the kidneys and extrarenal cells (Fig. 2). To investigate further, this study evaluated the effects of GW4869 (Fig. 2), a systemic inhibitor of small EV biogenesis [27, 29], on aortic VC. CKD C57BL/6JJcl mice fed an adenine-containing and high-Pi diet received intraperitoneal injections of 2.5 mg/kg GW4869 or its vehicle (7.5% DMSO in saline) every 2 days for 4 months. GW4869 almost entirely inhibited the increased mRNA transcription of osteogenic gene markers. This finding highlights the therapeutic potential of targeting cEVs by blocking MVB formation to prevent CKD-driven VC. Although GW4869 has shown renoprotective effects in vitro and in animal models, its clinical translation remains limited. Key challenges include the absence of human clinical trials, insufficient safety and pharmacokinetic data, and potential off-target effects due to its broad inhibition of neutral sphingomyelinase. In addition, efficient delivery to renal tissues and sustained suppression of EV release in vivo are unresolved. Future studies must address these challenges to realize the therapeutic potential of GW4869 in kidney disease.

We also determined the transcriptomic profiling of miRNAs in cEVs from CKD rodent models. miRNA array analyses in rats revealed the depletion of four miRNAs: miR-16-5p, miR-17 ~ 92 cluster-derived miR-17-5p/miR-20a-5p, and miR-106b-5p. This profile was replicated in CKD model mice and patients, in cEVs purified using polymer-based methods and sequential ultracentrifugation. In addition, we demonstrated that these miRNAs are protective against VC in vitro, and their lower expression was correlated with a lower estimated eGFR in 37 dialysis-independent CKD patients. Furthermore, we found that the expression levels of these miRNAs in cEVs were predictive of thoracic aortic calcification and coronary arterial calcification on CT scans among 35 patients who underwent this examination [57].

In silico and experimental analyses identified vascular endothelial growth factor A (VEGFA) as one of the primary target molecules of these miRNAs (Fig. 3). The VEGFA-VEGFR2-RUNX2 pathway is suppressed by miRNAs enriched in healthy cEVs. Still, this safeguard is disrupted by the depletion of protective miRNAs in CKD-derived cEVs. These findings suggest that cEVs may serve as novel key players, both therapeutic targets and minimally invasive biomarkers, in CKD.

A recent cross-sectional human study in Ref. [61] supported our findings, demonstrating the prognostic power of circulating total miR-16-5p extracted from the plasma of 132 patients undergoing maintenance hemodialysis. This study extracted total RNA from 200 μL of plasma using the TRIzol reagent. A one-quartile decrease in miR-16-5p expression was associated with an adjusted odds ratio of 5.336 (95% CI: 2.670–10.662) for a significant increase in the abdominal aortic calcification score. Another study demonstrated that miR-17-5p suppressed osteogenic VSMC differentiation by targeting transforming growth factor β receptor type II (TGF-β RII) in MOVAS cells. However, the pathophysiological context and isolation methods differed [62]. In this study, small EVs were purified from the supernatants of peritoneal macrophages exposed to high-glucose conditions using the Tim4-affinity method with the MagCapture™ Exosome Isolation Kit PS (FUJIFILM Wako Chemicals USA Corp).

As summarized in Table 2, the roles of miR-16-5p, miR-106b-5p, and the miR-17 cluster in CKD pathophysiology are context-dependent and complex. They generally show protective effects in CKD, whereas some AKI models report harmful roles. These variations likely reflect the kidney’s multicellular composition and the distinct mechanisms underlying AKI and CKD. Further research, including advanced methods to quantify functional miRNA transfer both with and without EV encapsulation, is essential to better understand intercellular communication between kidney and extrarenal cells.

## Conclusion

CKD cannot simply be defined as GFR decline, but as a complex systemic condition that accelerates aging and increases mortality, with CVD as the leading cause of death. Emerging evidence highlights the critical role of cEVs in the mediation of inter-organ communication, particularly between the kidneys and VSMCs. Our study demonstrated that CKD-derived cEVs, characterized by the depletion of protective microRNAs such as miR-16-5p, miR-17-5p, miR-20a-5p, and miR-106b-5p, promote VC by dysregulating the VEGFA-VEGFR2 signaling pathway. This pathological mechanism underscores the potential of cEVs as diagnostic biomarkers and therapeutic targets in CKD. Future research should focus on investigating the precise molecular mechanisms of cEV-mediated inter-organ communication and developing innovative strategies to restore the protective functions of cEVs. By leveraging these insights, kidney medicine could advance toward novel diagnostic and therapeutic platforms, ultimately improving outcomes for CKD patients.

## References

[1] Kalantar-Zadeh K, Jafar TH, Nitsch D, Neuen BL, Perkovic V. Chronic kidney disease. Lancet. 2021;398(10302):786–802. 10.1016/S0140-6736(21)00519-5. 34175022 10.1016/S0140-6736(21)00519-5

[2] Jager KJ, Kovesdy C, Langham R, Rosenberg M, Jha V, Zoccali C. A single number for advocacy and communication—worldwide more than 850 million individuals have kidney diseases. Nephrol Dial Transplant. 2019;34(11):1803–5. 10.1093/ndt/gfz174. 31566230 10.1093/ndt/gfz174

[3] Chesnaye NC, Ortiz A, Zoccali C, Stel VS, Jager KJ. The impact of population ageing on the burden of chronic kidney disease. Nat Rev Nephrol. 2024;20:569–85. 10.1038/s41581-024-00863-9. 39025992 10.1038/s41581-024-00863-9

[4] World Health Organization. Cardiovascular diseases. 2019. http://www.who.int/mediacentre/factsheets/fs317/en/.

[5] Ishigami J, Grams ME, Chang AR, Carrero JJ, Coresh J, Matsushita K. CKD and risk for hospitalization with infection: the atherosclerosis risk in communities (ARIC) study. Am J Kidney Dis. 2017;69(6):752–61. 10.1053/j.ajkd.2016.09.018. 27884474 10.1053/j.ajkd.2016.09.018 PMC5438909

[6] Scheppach JB, Coresh J, Wu A, Gottesman RF, Mosley TH, Knopman DS, et al. Albuminuria and estimated GFR as risk factors for dementia in midlife and older age: findings from the ARIC study. Am J Kidney Dis. 2020;76(6):775–83. 10.1053/j.ajkd.2020.03.015. 32428540 10.1053/j.ajkd.2020.03.015 PMC7669634

[7] Xu H, Garcia-Ptacek S, Trevisan M, Evans M, Lindholm B, Eriksdotter M, et al. Kidney function, kidney function decline, and the risk of dementia in older adults: a registry-based study. Neurology. 2021;96(24):e2956–65. 10.1212/WNL.0000000000012113. 33952656 10.1212/WNL.0000000000012113 PMC8253567

[8] Watanabe H, Enoki Y, Maruyama T. Sarcopenia in chronic kidney disease: factors, mechanisms, and therapeutic interventions. Biol Pharm Bull. 2019;42(9):1437–45. 10.1248/bpb.b19-00513. 31474705 10.1248/bpb.b19-00513

[9] Mandai S, Sato H, Iimori S, Naito S, Tanaka H, Ando F, et al. Nationwide in-hospital mortality following major fractures among hemodialysis patients and the general population: an observational cohort study. Bone. 2020;130:115122. 10.1016/j.bone.2019.115122. 31678496 10.1016/j.bone.2019.115122

[10] Tonelli M, Wiebe N, Manns BJ, Klarenbach SW, James MT, Ravani P, et al. Comparison of the complexity of patients seen by different medical subspecialists in a universal health care system. JAMA Netw Open. 2018;1(7):e184852. 10.1001/jamanetworkopen.2018.4852. 30646392 10.1001/jamanetworkopen.2018.4852 PMC6324421

[11] Elsayed EF, Tighiouart H, Griffith J, Weiner DE, Salem DN, Levey AS, et al. Cardiovascular disease and subsequent kidney disease. Arch Intern Med. 2007;167(11):1130–6. 10.1001/archinte.167.11.1130. 17563020 10.1001/archinte.167.11.1130

[12] Su G, Trevisan M, Ishigami J, Matsushita K, Stålsby Lundborg C, Carrero JJ. Short- and long-term outcomes after incident pneumonia in adults with chronic kidney disease: a time-dependent analysis from the Stockholm CREAtinine Measurement project. Nephrol Dial Transplant. 2020;35(11):1894–900. 10.1093/ndt/gfz119. 31219575 10.1093/ndt/gfz119 PMC7643674

[13] Nakano Y, Mandai S, Naito S, Fujiki T, Mori Y, Ando F, et al. Effect of osteosarcopenia on longitudinal mortality risk and chronic kidney disease progression in older adults. Bone. 2024;179:116975. 10.1016/j.bone.2023.116975. 37993037 10.1016/j.bone.2023.116975

[14] Tian YE, Cropley V, Maier AB, Lautenschlager NT, Breakspear M, Zalesky A. Heterogeneous aging across multiple organ systems and prediction of chronic disease and mortality. Nat Med. 2023;29(5):1221–31. 10.1038/s41591-023-02296-6. 37024597 10.1038/s41591-023-02296-6

[15] Kooman JP, Kotanko P, Schols AMWJ, Shiels PG, Stenvinkel P. Chronic kidney disease and premature ageing. Nat Rev Nephrol. 2014;10(12):732–42. 10.1038/nrneph.2014.185. 25287433 10.1038/nrneph.2014.185

[16] Welsh JA, Goberdhan DCI, O’Driscoll L, Buzas EI, Blenkiron C, Bussolati B, et al. Minimal information for studies of extracellular vesicles (MISEV2023): from basic to advanced approaches. J Extracell Vesicles. 2024;13(2):e12404. 10.1002/jev2.12404. 38326288 10.1002/jev2.12404 PMC10850029

[17] Wolf P. The nature and significance of platelet products in human plasma. Br J Haematol. 1967;13(3):269–88. 10.1111/j.1365-2141.1967.tb08741.x. 6025241 10.1111/j.1365-2141.1967.tb08741.x

[18] Trams EG, Lauter CJ, Salem N Jr, Heine U. Exfoliation of membrane ecto-enzymes in the form of micro-vesicles. Biochim Biophys Acta. 1981;645(1):63–70. 10.1016/0005-2736(81)90512-5 6266476 10.1016/0005-2736(81)90512-5

[19] Harding C, Heuser J, Stahl P. Receptor-mediated endocytosis of transferrin and recycling of the transferrin receptor in rat reticulocytes. J Cell Biol. 1983;97(2):329–39. 10.1083/jcb.97.2.329. 6309857 10.1083/jcb.97.2.329 PMC2112509

[20] Pan BT, Teng K, Wu C, Adam M, Johnstone RM. Electron microscopic evidence for externalization of the transferrin receptor in vesicular form in sheep reticulocytes. J Cell Biol. 1985;101(3):942–8. 10.1083/jcb.101.3.942. 2993317 10.1083/jcb.101.3.942 PMC2113705

[21] Johnstone RM, Adam M, Hammond JR, Orr L, Turbide C, et al. Vesicle formation during reticulocyte maturation. Association of plasma membrane activities with released vesicles (exosomes). J Biol Chem. 1987;262(19):9412–20. 10.1016/S0021-9258(18)48095-7. 3597417

[22] Witwer KW. Minimal information for studies of extracellular vesicles 2023: relevance to cell and gene therapies. Cytotherapy. 2024;26(10):1119–21. 10.1016/j.jcyt.2024.05.018. 39046387 10.1016/j.jcyt.2024.05.018

[23] van Niel G, D’Angelo G, Raposo G. Shedding light on the cell biology of extracellular vesicles. Nat Rev Mol Cell Biol. 2018;19:213–28. 10.1038/nrm.2017.125. 29339798 10.1038/nrm.2017.125

[24] Teng F, Fussenegger M. Shedding light on extracellular vesicle biogenesis and bioengineering. Adv Sci. 2020;8(1):2003505. 10.1002/advs.202003505. 10.1002/advs.202003505 PMC7788585 33437589

[25] Korbei B. Ubiquitination of the ubiquitin-binding machinery: how early ESCRT components are controlled. Essays Biochem. 2022;66(2):169–77. 10.1042/EBC20210042. 35352804 10.1042/EBC20210042 PMC9400068

[26] Trajkovic K, Hsu C, Chiantia S, Rajendran L, Wenzel D, Wieland F, et al. Ceramide triggers budding of exosome vesicles into multivesicular endosomes. Science. 2008;319(5867):1244–7. 10.1126/science.1153124 18309083 10.1126/science.1153124

[27] Airola MV, Shanbhogue P, Shamseddine AA, Guja KE, Senkal CE, Maini R, et al. Structure of human nSMase2 reveals an interdomain allosteric activation mechanism for ceramide generation. Proc Natl Acad Sci U S A. 2017;114(28):E5549-E5558. 10.1073/pnas.1705134114. 28652336 10.1073/pnas.1705134114 PMC5514751

[28] Hyung S, Kim J, Yu C, Jung H, Hong J. Neuroprotective effect of glial cell-derived exosomes on neurons. Immunotherapy. 2019;5:156. 10.2217/imt-2018-0156.

[29] Asai H, Ikezu S, Tsunoda S, Medalla M, Luebke J, Haydar T, et al. Depletion of microglia and inhibition of exosome synthesis halt tau propagation. Nat Neurosci. 2015;18:1584–93. 10.1038/nn.4132. 26436904 10.1038/nn.4132 PMC4694577

[30] Ostrowski M, Carmo NB, Krumeich S, Fanget I, Raposo G, Savina A, et al. Rab27a and Rab27b control different steps of the exosome secretion pathway. Nat Cell Biol. 2010;12(1):19–30. 10.1038/ncb2000. 19966785 10.1038/ncb2000

[31] Yuyama K, Sun H, Mikami D, Mioka T, Mukai K, Igarashi Y. Lysosomal-associated transmembrane protein 4B regulates ceramide-induced exosome release. FASEB J. 2020;34(12):16022–33. 10.1096/fj.202001599R. 33090522 10.1096/fj.202001599R

[32] Lauwers E, Wang YC, Gallardo R, Van der Kant R, Michiels E, Swerts J, et al. Hsp90 mediates membrane deformation and exosome release. Mol Cell. 2018;71(5):689-702.e9. 10.1016/j.molcel.2018.07.016. 30193096 10.1016/j.molcel.2018.07.016

[33] Valadi H, Ekström K, Bossios A, Sjöstrand M, Lee JJ, Lötvall JO. Exosome-mediated transfer of mRNAs and microRNAs is a novel mechanism of genetic exchange between cells. Nat Cell Biol. 2007;9(6):654–9. 10.1038/ncb1596 17486113 10.1038/ncb1596

[34] Alexiou P, Maragkakis M, Papadopoulos GL, Reczko M, Hatzigeorgiou AG. Lost in translation: an assessment and perspective for computational microRNA target identification. Bioinformatics. 2009;25(23):3049–55. 10.1093/bioinformatics/btp565 19789267 10.1093/bioinformatics/btp565

[35] Zomer A, Maynard C, Verweij FJ, Kamermans A, Schäfer R, Beerling E, et al. In vivo imaging reveals extracellular vesicle-mediated phenocopying of metastatic behavior. Cell. 2015;161(5):1046–57. 10.1016/j.cell.2015.04.042 26000481 10.1016/j.cell.2015.04.042 PMC4448148

[36] de Jong OG, Murphy DE, Mäger I, Willms E, Garcia-Guerra A, Gitz-Francois JJ, et al. A CRISPR-Cas9-based reporter system for single-cell detection of extracellular vesicle-mediated functional transfer of RNA. Nat Commun. 2020;11(1):1113. 10.1038/s41467-020-14977-8. 32111843 10.1038/s41467-020-14977-8 PMC7048928

[37] Erdbrügger U, Blijdorp CJ, Bijnsdorp IV, Borras FE, Burger D, Bussolati B, et al. Urinary extracellular vesicles: a position paper by the Urine Task Force of the International Society for Extracellular Vesicles. J Extracell Vesicles. 2021;10(7):e12093. 10.1002/jev2.12093. 34035881 10.1002/jev2.12093 PMC8138533

[38] Svenningsen P, Sabaratnam R, Jensen BL. Urinary extracellular vesicles: origin, role as intercellular messengers and biomarkers; efficient sorting and potential treatment options. Acta Physiol (Oxf). 2020;228(1):e13346. 10.1111/apha.13346. 31334916 10.1111/apha.13346

[39] Street JM, Barran PE, Mackay CL, Weidt S, Balmforth C, Walsh TS, et al. Exosomal transmission of functional aquaporin 2 in kidney cortical collecting duct cells. J Physiol. 2011;589(24):6119–27. 10.1113/jphysiol.2011.220277. 22025668 10.1113/jphysiol.2011.220277 PMC3286690

[40] Gildea JJ, Seaton JE, Victor KG, Reyes CM, Bigler Wang D, Pettigrew AC, et al. Exosomal transfer from human renal proximal tubule cells to distal tubule and collecting duct cells. Clin Biochem. 2014;47(1–2):89–94. 10.1016/j.clinbiochem.2013.10.021. 24976626 10.1016/j.clinbiochem.2014.06.018 PMC5384615

[41] Hunter RW, Kumar S, Coward RJM, Buck AH, Dear JW. Extracellular RNA moves from the glomerulus to the renal tubule. Preprint at bioRxiv. 2021. 10.1101/2021.06.15.448584.

[42] Lv LL, Cao YH, Ni HF, Xu M, Liu D, Liu H, et al. MicroRNA-29c in urinary exosome/microvesicle as a biomarker of renal fibrosis. Am J Physiol Renal Physiol. 2013;305(8):F1220–7. 10.1152/ajprenal.00148.2013. 23946286 10.1152/ajprenal.00148.2013

[43] Khurana R, Ranches G, Schafferer S, Lukasser M, Rudnicki M, Mayer G, et al. Identification of urinary exosomal noncoding RNAs as novel biomarkers in chronic kidney disease. RNA. 2017;23:142–52. 10.1261/rna.058834.116 27872161 10.1261/rna.058834.116 PMC5238789

[44] Min QH, Chen XM, Zou YQ, Zhang J, Li J, Wang Y, et al. Differential expression of urinary exosomal microRNAs in IgA nephropathy. J Clin Lab Anal. 2018;32(2):e22226. 10.1002/jcla.22226. 28383146 10.1002/jcla.22226 PMC6816951

[45] Barutta F, Tricarico M, Corbelli A, Annaratone L, Pinach S, Grimaldi S, et al. Urinary exosomal microRNAs in incipient diabetic nephropathy. PLoS ONE. 2013;8:e73798. 10.1371/journal.pone.0073798. 24223694 10.1371/journal.pone.0073798 PMC3817183

[46] Blijdorp CJ, Hartjes TA, Wei KY, van Heugten MH, Bovée DM, Budde RPJ, et al. Nephron mass determines the excretion rate of urinary extracellular vesicles. J Extracell Vesicles. 2022;11(1):e12181. 10.1002/jev2.12181 35064766 10.1002/jev2.12181 PMC8783354

[47] Claridge B, Rai A, Fang H, Matsumoto A, Luo J, McMullen JR, et al. Proteome characterisation of extracellular vesicles isolated from heart. Proteomics. 2021;21(13–14):2100026. 10.1002/pmic.202100026. 10.1002/pmic.202100026 33861516

[48] Salomon C, Torres MJ, Kobayashi M, Scholz-Romero K, Sobrevia L, Dobierzewska A, et al. A gestational profile of placental exosomes in maternal plasma and their effects on endothelial cell migration. PLoS ONE. 2014;9(6):e98667. 10.1371/journal.pone.0098667. 24905832 10.1371/journal.pone.0098667 PMC4048215

[49] Manojlovic M, Juto A, Jonasdottir A, Colic J, Vojinovic J, Nordin A, et al. Microparticles expressing myeloperoxidase as potential biomarkers in anti-neutrophil cytoplasmic antibody (ANCA)-associated vasculitides (AAV). J Mol Med. 2020;98:1279–86. 10.1007/s00109-020-01936-4. 32734361 10.1007/s00109-020-01955-2 PMC7447662

[50] Miao D, Ma TT, Chen M, Zhao MH. Platelets release proinflammatory microparticles in anti-neutrophil cytoplasmic antibody-associated vasculitis. Rheumatology. 2019;58(8):1432–42. 10.1093/rheumatology/kez093. 10.1093/rheumatology/kez044 30843591

[51] Saejong S, Townamchai N, Somparn P, Tangtanatakul P, Ondee T, Hirankarn N, et al. MicroRNA-21 in plasma exosome, but not from whole plasma, as a biomarker for the severe interstitial fibrosis and tubular atrophy (IF/TA) in post-renal transplantation. Asian Pac J Allergy Immunol. 2022;40(1):94–102. 10.12932/AP-101019-0656. 32563236 10.12932/AP-101019-0656

[52] Kim H, Bae YU, Jeon JS, Noh H, Park HK, Byun DW, et al. The circulating exosomal microRNAs related to albuminuria in patients with diabetic nephropathy. J transl med. 2019;17:1–11. 10.1186/s12967-019-1974-8. 31331349 10.1186/s12967-019-1983-3 PMC6647278

[53] Ramezani A, Devaney JM, Cohen S, Wing MR, Scott R, Knoblach S, et al. Circulating and urinary microRNA profile in focal segmental glomerulosclerosis: a pilot study. Eur J Clin Invest. 2015;45(4):394–404. 10.1111/eci.12425. 25682967 10.1111/eci.12420 PMC4903079

[54] Kapustin AN, Shanahan CM, Schurgers LJ. Prothrombin loading of vascular smooth muscle cell–derived exosomes regulates coagulation and calcification. Arterioscler Thromb Vasc Biol. 2017;37(3):e22–32. 10.1161/ATVBAHA.116.308648. 28104608 10.1161/ATVBAHA.116.308886

[55] Sage AP, Tintut Y, Demer LL. Regulatory mechanisms in vascular calcification. Nat Rev Cardiol. 2010;7:528–36. 10.1038/nrcardio.2010.115. 20664518 10.1038/nrcardio.2010.115 PMC3014092

[56] Durham AL, Speer MY, Scatena M, Giachelli CM, Shanahan CM. Role of smooth muscle cells in vascular calcification: implications in atherosclerosis and arterial stiffness. Cardiovasc Res. 2018;114(4):590–600. 10.1093/cvr/cvy010. 29514202 10.1093/cvr/cvy010 PMC5852633

[57] Koide T, Mandai S, Kitaoka R, Matsuki H, Chiga M, Yamamoto K, et al. Circulating extracellular vesicle-propagated microRNA signature as a vascular calcification factor in chronic kidney disease. Circ Res. 2023;132(4):415–31. 10.1161/CIRCRESAHA.122.321939. 36700539 10.1161/CIRCRESAHA.122.321939

[58] Cazaña-Pérez V, Cidad P, Donate-Correa J, Martín-Núñez E, López-López JR, Pérez-García MT, et al. Phenotypic modulation of cultured primary human aortic vascular smooth muscle cells by uremic serum. Front Physiol. 2018;9:89. 10.3389/fphys.2018.00089. 29483881 10.3389/fphys.2018.00089 PMC5816230

[59] Sahoo S, Adamiak M, Mathiyalagan P, Kenneweg F, Kafert-Kasting S, Thum T. Therapeutic and diagnostic translation of extracellular vesicles in cardiovascular diseases: roadmap to the clinic. Circulation. 2021;143:1426–49. 10.1161/circulationaha.120.049254. 33819075 10.1161/CIRCULATIONAHA.120.049254 PMC8021236

[60] Bhagirath D, Yang TL, Bucay N, Sekhon K, Majid S, Shahryari V, et al. MicroRNA-1246 is an exosomal biomarker for aggressive prostate cancer. Cancer Res. 2018;78(7):1833–44. 10.1158/0008-5472.CAN-17-2069. 29437039 10.1158/0008-5472.CAN-17-2069 PMC5890910

[61] Liang Q, Fu C, Liu Y, Liu W, Guo W. Association of plasma microRNA-16-5p and abdominal aortic calcification in maintenance hemodialysis patients. Ren Fail. 2024;46(2):2368091. 10.1080/0886022X.2024.2368091. 39049724 10.1080/0886022X.2024.2368091 PMC11275526

[62] Baba I, Matoba T, Katsuki S, Koga J-I, Kawahara T, Kimura M, et al. EVs-miR-17-5p attenuates the osteogenic differentiation of vascular smooth muscle cells potentially via inhibition of TGF-β signaling under high glucose conditions. Sci Rep. 2024;14(1):16323. 10.1038/s41598-024-16323-7. 39009669 10.1038/s41598-024-67006-9 PMC11251274

[63] Lv CY, Zhao ZY, Yang TL, Wang YL, Li B, Lv J, et al. Liquid biopsy biomarkers of renal interstitial fibrosis based on urinary exosome. Exp Mol Pathol. 2018;105(2):223–8. 10.1016/j.yexmp.2018.08.004. 30121168 10.1016/j.yexmp.2018.08.004

[64] Lv CY, Ding WJ, Wang YL, Zhao ZY, Li JH, Chen Y, et al. A PEG-based method for the isolation of urinary exosomes and its application in renal fibrosis diagnostics using cargo miR-29c and miR-21 analysis. Int Urol Nephrol. 2018;50(5):973–82. 10.1007/s11255-017-1779-4. 29330775 10.1007/s11255-017-1779-4

[65] Jia Y, Guan M, Zheng Z, Zhang Q, Tang C, Xu W, et al. miRNAs in urine extracellular vesicles as predictors of early-stage diabetic nephropathy. J Diabetes Res. 2016;2016:7932765. 10.1155/2016/7932765. 10.1155/2016/7932765 PMC4749815 26942205

[66] Liu D, Chen R, Ni H, Liu H. miR-181a improved renal inflammation by targeting TNF-α in a diabetic nephropathy animal model. Nephron. 2022;146(6):637–46. 10.1159/000525050. 35810742 10.1159/000525050

[67] Liu L, Pang XL, Shang WJ, Xie HC, Wang JX, Feng GW. Over-expressed microRNA-181a reduces glomerular sclerosis and renal tubular epithelial injury in rats with chronic kidney disease via down-regulation of the TLR/NF-κB pathway by bindingto CRY1. Mol Med. 2018;24(1):49. 10.1186/s10020-018-0045-2. 30241461 10.1186/s10020-018-0045-2 PMC6145098

[68] Eissa S, Matboli M, Aboushahba R, Bekhet MM, Soliman Y. Urinary exosomal microRNA panel unravels novel biomarkers for diagnosis of type 2 diabetic kidney disease. J Diabetes Complications. 2016;30(8):1585–92. 10.1016/j.jdiacomp.2016.07.012. 27475263 10.1016/j.jdiacomp.2016.07.012

[69] Solé C, Moliné T, Vidal M, Ordi-Ros J, Cortés-Hernández J. An exosomal urinary miRNA signature for early diagnosis of renal fibrosis in lupus nephritis. Cells. 2019;8(8):773. 10.3390/cells8080773. 31349698 10.3390/cells8080773 PMC6721515

[70] Xie JX, Fan X, Drummond CA, Majumder R, Xie Y, Chen T, et al. MicroRNA profiling in kidney disease: Plasma versus plasma-derived exosomes. Gene. 2017;627:1–8. 10.1016/j.gene.2017.06.003. 28587849 10.1016/j.gene.2017.06.003 PMC5534180

[71] Park S, Kim OH, Lee K, Park IB, Kim NH, Moon S, et al. Plasma and urinary extracellular vesicle microRNAs and their related pathways in diabetic kidney disease. Genomics. 2022;114(4):110407. 10.1016/j.ygeno.2022.110407. 35716820 10.1016/j.ygeno.2022.110407

[72] Li H, Duan J, Zhang T, Fu Y, Xu Y, Miao H, et al. miR-16-5p aggravates sepsis-associated acute kidney injury by inducing apoptosis. Ren Fail. 2024;46(1):2322688. 10.1080/0886022X.2024.2322688. 38445373 10.1080/0886022X.2024.2322688 PMC10919310

[73] Chen HH, Lan YF, Li HF, Cheng CF, Lai PF, Li WH, et al. Urinary miR-16 transactivated by C/EBPβ reduces kidney function after ischemia/reperfusion-induced injury. Sci Rep. 2016;6:27945. 10.1038/srep27945. 27297958 10.1038/srep27945 PMC4906401

[74] Duan YR, Chen BP, Chen F, Yang SX, Zhu CY, Ma YL, et al. Exosomal microRNA-16-5p from human urine-derived stem cells ameliorates diabetic nephropathy through protection of podocyte. J Cell Mol Med. 2021;25(23):10798–813. 10.1111/jcmm.14558. 31568645 10.1111/jcmm.14558 PMC8642687

[75] Hao J, Wei Q, Mei S, Li L, Su Y, Mei C, et al. Induction of microRNA-17-5p by p53 protects against renal ischemia-reperfusion injury by targeting death receptor 6. Kidney Int. 2017;91(1):106–118. 10.1016/j.kint.2016.07.017 27622990 10.1016/j.kint.2016.07.017 PMC5179285

[76] Suda A, Liu X, Ozbaki Yagan N, Tan R, Ho J, Butterworth M. Renal epithelial-derived miR-17~92 inhibits STAT3 signaling, serving a sex-specific and renoprotective role in the development of AKI. Physiology. 2024;39(S1):1516. 10.1152/physiol.2024.39.S1.1516.

